# Psychological Well-Being and Burnout in University Faculty: The Mediating Role of Emotional Exhaustion

**DOI:** 10.62641/aep.v54i4.2346

**Published:** 2026-08-15

**Authors:** María J. García-Rubio, Laura Viqueira Gutiérrez, Sandra Hoyos, Paloma López-Hernández, Irene Cano-López, Alejandro Cano-Villagrasa

**Affiliations:** ^1^Faculty of Health Sciences, Universidad Internacional de Valencia, 46002 Valencia, Spain; ^2^Department of Neuroscience and Learning, Catholic University of Uruguay, 11600 Montevideo, Uruguay; ^3^Institut d’Investigació en Psicologia dels Recursos Humans, del Desenvolupament Organitzacional i de la Qualitat de Vida Laboral (IDOCAL)/Department of Psychobiology, Psychology Center, Universitat de València, 46010 Valencia, Spain

**Keywords:** burnout, emotional exhaustion, psychological well-being, university faculty, personal accomplishment

## Abstract

**Background:**

To examine the relationship between psychological well-being and burnout dimensions among university faculty members, as well as the mediating role of emotional exhaustion in the association between psychological well-being and professional accomplishment.

**Methods:**

A cross-sectional study was conducted with 597 university faculty members from several Spanish universities. Participants completed the Spanish adaptation of Ryff’s Psychological Well-Being Scales and the emotional exhaustion and personal accomplishment subscales of the Maslach Burnout Inventory. Correlation, regression, and mediation analyses were performed.

**Results:**

All dimensions of psychological well-being were negatively associated with emotional exhaustion and positively associated with professional accomplishment. Environmental mastery emerged as the strongest predictor of lower emotional exhaustion (β = −0.405, *p* < 0.001) and higher professional accomplishment (β = 0.263, *p* < 0.001). Mediation analyses indicated that emotional exhaustion partially mediated the relationship between psychological well-being and professional accomplishment.

**Conclusions:**

Psychological well-being may function as a protective personal resource associated with lower levels of burnout among university faculty members. In particular, environmental mastery may represent a key psychological resource associated with lower emotional exhaustion and greater professional accomplishment in higher education settings. Interventions aimed at strengthening psychological well-being and reducing emotional exhaustion may contribute to improving occupational mental health in academic professionals.

## Introduction

Burnout among university faculty has become a major occupational and mental health concern in higher education. The World Health Organization recognizes burnout in the ICD-11 as an occupational phenomenon resulting from chronic workplace stress that has not been successfully managed, whereas the classical model proposed by Maslach conceptualizes it through emotional exhaustion, depersonalization or cynicism, and reduced professional accomplishment [1, 2]. In university settings, this syndrome is especially relevant because academic work extends far beyond teaching activities and includes research productivity, grant acquisition, administrative responsibilities, student supervision, continuous evaluation, and increasing institutional demands. Consequently, university faculty are frequently exposed to sustained psychosocial stressors that may negatively affect both professional functioning and psychological adjustment [3].

Research conducted in higher education consistently indicates elevated levels of stress and burnout among academic staff [4]. A systematic review including more than 43,000 university faculty members identified workload, limited institutional support, and work-related conflict as the most consistent predictors of burnout in higher education [5]. More recent studies continue to report significant associations between burnout and factors such as workload, long working hours, work-life imbalance, and reduced well-being [6]. Beyond its impact on individual mental health, burnout has also been associated with absenteeism, reduced productivity, lower job satisfaction, and increased turnover intentions [2, 4]. Furthermore, evidence suggests that teacher burnout may negatively affect students’ academic performance and motivational outcomes, highlighting its broader implications for educational quality and institutional functioning [7].

Given these consequences, recent research has increasingly focused not only on occupational risk factors but also on psychological resources that may protect university faculty from burnout. Among these resources, Ryff’s eudaimonic model of psychological well-being offers a particularly useful framework [8, 9]. Unlike approaches centered exclusively on subjective happiness or the absence of psychopathology, Ryff conceptualized psychological well-being as positive psychological functioning and human development. This multidimensional model includes self-acceptance, positive relations with others, autonomy, environmental mastery, purpose in life, and personal growth. In academic contexts, these dimensions are especially relevant because they reflect psychological capacities linked to self-regulation, interpersonal functioning, effective management of complex demands, and continuous personal development. The Spanish adaptation developed by Díaz *et al*. [10] demonstrated adequate psychometric properties in Spanish-speaking populations and has been widely used in applied psychological research [10, 11].

Previous research has shown significant associations between psychological well-being and burnout in educational professionals. Lower levels of autonomy, environmental mastery, personal growth, positive relationships, and self-acceptance have been associated with greater emotional exhaustion and lower professional accomplishment [12]. Among these dimensions, environmental mastery appears particularly relevant in teaching professionals because it reflects the perceived ability to effectively manage occupational demands and organizational challenges [13]. Likewise, interpersonal functioning and emotional adjustment have been identified as important protective factors against psychological distress and occupational exhaustion in teachers [14]. Altogether, these findings suggest that psychological well-being may operate as a protective factor against burnout, although not all dimensions necessarily contribute equally. From the perspective of the Job Demands–Resources (JD-R) model [15], psychological well-being may be understood as a personal resource that facilitates adaptation to demanding occupational contexts. Personal resources are aspects of the self that are generally linked to resilience, effective coping, and the ability to influence one’s environment successfully. Accordingly, psychological well-being may be conceptualized as a constellation of personal resources that support adaptive functioning and facilitate the management of occupational demands. In this framework, dimensions of psychological well-being such as environmental mastery, purpose in life, and self-acceptance may help individuals manage occupational demands more effectively and reduce vulnerability to burnout. Among these dimensions, environmental mastery may be especially relevant in university settings characterized by multiple and often competing professional demands. Because it reflects the perceived ability to manage responsibilities, organize resources, and effectively navigate complex environments, environmental mastery may be more directly related to the day-to-day challenges of academic work than other dimensions of psychological well-being. Consequently, it was expected to show the strongest association with burnout indicators.

Among the dimensions of burnout, emotional exhaustion appears to occupy a central role. Maslach and Leiter described emotional exhaustion as the core component of burnout and the most direct manifestation of chronic occupational stress [2]. In teachers, emotional exhaustion has been associated with lower work engagement, reduced instructional quality, poorer mental health, and decreased professional satisfaction [14]. More recent research suggests that emotional exhaustion may function as a mechanism linking occupational stressors with poorer psychological adjustment and lower professional satisfaction in university faculty. For example, Xu and Wang [16] found that emotional exhaustion mediated the relationship between occupational stress and life satisfaction among early-career university faculty. Similarly, previous mediation studies suggest that positive psychological resources may reduce burnout and contribute to greater professional efficacy and adjustment [17].

Despite growing evidence regarding the relationship between psychological well-being and burnout, several gaps remain. First, many studies have relied on global indicators of well-being or related constructs such as flourishing or psychological capital, whereas fewer investigations have specifically examined Ryff’s multidimensional model. Second, university faculty remain comparatively underrepresented in this literature, as much of the available evidence derives from school teachers or mixed educational samples. Finally, limited attention has been paid to the potential mediating role of emotional exhaustion in the relationship between psychological well-being and professional accomplishment in higher education settings.

Previous studies have also explored mediating and protective mechanisms linking psychological resources and burnout among educational and occupational populations [18]. Research conducted with Spanish university faculty identified indirect relationships between psychological variables and burnout indicators, supporting the relevance of mediation processes in explaining occupational adjustment [19]. In educational settings, psychological contract theory has also been used to explain burnout, suggesting that the fulfillment of organizational expectations and perceived reciprocity between teachers and institutions may function as protective factors against emotional exhaustion, whereas violations of these expectations are associated with poorer occupational adjustment and greater vulnerability to burnout [20]. Similarly, recent evidence has shown that psychological resilience may partially mediate the relationship between psychological distress and burnout, acting as a protective resource against emotional exhaustion and occupational maladjustment [21]. Collectively, these findings suggest that both personal resources and organizational factors may exert their influence on occupational functioning through intermediate psychological processes. The present study extends this line of research by examining emotional exhaustion as a potential mediator between psychological well-being and professional accomplishment.

Therefore, the present study examined the relationship between psychological well-being and burnout dimensions among university faculty members, with particular attention to the contribution of Ryff’s psychological well-being dimensions and the mediating role of emotional exhaustion in professional accomplishment. Based on previous literature and the JD-R framework, environmental mastery was expected to show the strongest association with burnout indicators. Environmental mastery reflects the perceived ability to manage environmental demands, organize responsibilities, and maintain control over complex situations. Given the multiple and often competing demands associated with academic work, this dimension may be especially relevant for university faculty members. Specifically, three hypotheses were proposed: (H1) higher psychological well-being would be associated with lower emotional exhaustion and greater professional accomplishment; (H2) environmental mastery would show the strongest association with burnout indicators; and (H3) emotional exhaustion would partially mediate the relationship between psychological well-being and professional accomplishment.

## Materials and Methods

### Participants

The sample consisted of 597 university faculty members from several Spanish universities. Detailed sociodemographic and occupational characteristics of the participants are presented in Table 1.

**Table 1.** **Sociodemographic characteristics of the participants**.

Variable	n (%)/M (SD)
Age (years)	50.91 (11.93)
Teaching experience (years)	19.56 (12.73)
Men	317 (53.1%)
Women	274 (45.9%)
Prefer not to answer	6 (1.0%)
Stable partner	499 (83.6%)
No stable partner	98 (16.4%)
With children	398 (66.7%)
Without children	199 (33.3%)
Public university	569 (95.3%)
Private university	28 (4.7%)

Note: Values are presented as frequencies and percentages or means and standard deviations (SD).

### Procedure

Participants were recruited through non-probabilistic convenience sampling. Faculty members from several Spanish universities were invited to participate through the distribution of an online questionnaire hosted on Microsoft Forms. The survey link was disseminated through institutional contacts and academic networks across the participating universities. The participating institutions were located in different regions of Spain, thereby increasing the geographical diversity of the sample. The recruitment and data collection process was coordinated by members of the research team. Data collection was conducted between September 2025 and February 2026. Participation was voluntary, anonymous, and no incentives were provided. Because the survey was distributed through institutional channels and academic networks, the exact response rate could not be calculated. Participation depended on voluntary self-selection.

Before completing the questionnaire, participants were informed about the aims of the study, the voluntary nature of participation, and the confidentiality and anonymity of their responses. All participants were required to read an information sheet and provide informed consent electronically before accessing the survey.

The questionnaire included an adapted version of Ryff’s Psychological Well-Being Scales, the emotional exhaustion and personal accomplishment subscales of the Maslach Burnout Inventory (MBI), and a sociodemographic questionnaire. Completion of the survey required approximately 15–20 minutes. The study was conducted in accordance with the ethical principles of the Declaration of Helsinki and was approved by the Research and Teaching Ethics Committee of the University of A Coruña (CEID-UDC; Approval Code: 2025-029). In addition, the study was reported following the Strengthening the Reporting of Observational Studies in Epidemiology (STROBE) guidelines [22].

### Instruments

Psychological well-being was assessed using an adapted version of the Spanish adaptation of Ryff’s Psychological Well-Being Scales developed by Díaz *et al*. [10]. Although the original Spanish adaptation comprises 39 items assessing six dimensions of psychological well-being, the present study focused on five dimensions: self-acceptance, positive relations, autonomy, environmental mastery, and personal growth. The Purpose in Life dimension was not included because the primary objective of the study was to examine psychological resources more directly associated with occupational functioning, burnout, and professional accomplishment among university faculty members. According to Ryff’s eudaimonic model, the Purpose in Life dimension primarily reflects broader aspects of existential meaning, life goals, and life direction [8, 23]. In contrast, the selected dimensions represent psychological resources more directly related to adaptive functioning, self-regulation, interpersonal relationships, and effective interaction with the work environment, all of which have been identified as relevant factors in occupational well-being and burnout research [5, 18]. Therefore, these five dimensions were selected to operationalize psychological well-being in accordance with the specific objectives of the present study. Participants responded using a Likert-type scale ranging from 1 (“strongly disagree”) to 6 (“strongly agree”). For the mediation analysis, a composite psychological well-being score was calculated by summing the scores of the five selected dimensions. Higher scores indicated greater psychological well-being. Previous studies reported adequate internal consistency for the assessed dimensions, with Cronbach’s alpha coefficients ranging from α = 0.68 to α = 0.83. In the present sample, Cronbach’s alpha coefficients were α = 0.857 for self-acceptance, α = 0.830 for positive relations, α = 0.762 for autonomy, α = 0.774 for environmental mastery, and α = 0.718 for personal growth. The composite psychological well-being score also showed good internal consistency (Cronbach’s α = 0.817).

Burnout was assessed using the Spanish version of the MBI, adapted by Gil-Monte and Peiró [24]. The original instrument consists of 22 items distributed across three dimensions: emotional exhaustion, depersonalization, and personal accomplishment at work. In the present study, analyses focused on the emotional exhaustion and personal accomplishment subscales because emotional exhaustion is widely considered the core dimension of burnout and personal accomplishment reflects occupational functioning and professional efficacy. Depersonalization was not included because the present study specifically aimed to examine the relationship between psychological well-being, emotional depletion, and professional accomplishment among university faculty members. This decision was also based on the study’s focus on the energetic and motivational components of burnout. Nevertheless, the exclusion of depersonalization may limit the comprehensiveness of the burnout construct and should be considered when interpreting the findings. Participants responded using a Likert-type scale ranging from 0 (“never”) to 6 (“every day”), indicating the frequency with which they experienced each situation during the last year. Previous studies reported adequate reliability for the emotional exhaustion (α = 0.86) and personal accomplishment (α = 0.76) subscales. In the present sample, Cronbach’s alpha coefficients were α = 0.909 for emotional exhaustion and α = 0.778 for personal accomplishment at work.

Additionally, a sociodemographic questionnaire was administered to gather information about participants’ personal and professional characteristics. Specifically, data were collected regarding sex, age, relationship status, parenthood, university affiliation, type of university (public or private), and years of teaching experience. These variables were included to examine possible differences in psychological well-being and burnout according to demographic and occupational factors.

### Statistical Analyses

Data analyses were conducted using IBM SPSS Statistics, version 29 (IBM Corp., Armonk, NY, USA). First, descriptive analyses were performed to summarize sociodemographic characteristics and the main study variables, including psychological well-being and burnout dimensions. The distribution of continuous variables was assessed using the Kolmogorov–Smirnov and Shapiro–Wilk tests, together with the examination of skewness, kurtosis, Q–Q plots, and boxplots. Although the normality tests were statistically significant, skewness and kurtosis values indicated only minor departures from normality. Given the large sample size and the approximately normal distribution of the variables, parametric statistical procedures were considered appropriate.

Pearson correlation analyses were computed to examine associations among the dimensions of psychological well-being, burnout variables, age, and teaching experience. Group comparisons based on dichotomous sociodemographic variables (sex, relationship status, parenthood, and type of university affiliation) were conducted using independent-samples *t*-tests to identify potential differences in psychological well-being dimensions.

Subsequently, multiple linear regression analyses were performed to examine the predictive contribution of the psychological well-being dimensions to emotional exhaustion and personal accomplishment at work. Emotional exhaustion and personal accomplishment were included as dependent variables, whereas the dimensions of psychological well-being were entered as predictors. Prior to conducting the regression analyses, assumptions of linearity, homoscedasticity, normality of residuals, and multicollinearity were examined.

Finally, mediation analyses were conducted to examine whether emotional exhaustion mediated the relationship between the composite psychological well-being score and personal accomplishment at work. A composite psychological well-being score was calculated by summing the scores of the five selected dimensions of psychological well-being (self-acceptance, positive relations, autonomy, environmental mastery, and personal growth). Mediation analyses were performed using the PROCESS macro for SPSS (Model 4) with 5000 bootstrap samples. The composite psychological well-being score was included as the independent variable, emotional exhaustion as the mediator, and personal accomplishment at work as the dependent variable. The indirect effect was considered statistically significant when the 95% bootstrap confidence interval did not include zero. Statistical significance was established at *p* < 0.05 for all analyses.

The datasets generated and analyzed during the current study are not publicly available due to ethical restrictions and participant confidentiality requirements established by the Research Ethics Committee. Data may be available from the corresponding author upon reasonable request and subject to approval by the relevant ethics authorities.

## Results

### Participant Characteristics

Sociodemographic and occupational characteristics of the participants are presented in Table 1. The sample consisted of 597 university faculty members from several Spanish universities. Participants ranged in age from 24 to 76 years and had between 1 and 54 years of teaching experience. Most participants were affiliated with public universities.

### Descriptive Statistics and Correlations Among Study Variables

Descriptive statistics and correlations among psychological well-being dimensions, the composite psychological well-being score, and burnout variables are presented in Table 2.

**Table 2.** **Descriptive statistics and correlations among psychological well-being dimensions, the composite psychological well-being score, and burnout variables**.

Variable	M	SD	1	2	3	4	5	6	7	8
1. Self-acceptance	27.85	5.19	—							
2. Positive relations	27.71	6.17	0.49**	—						
3. Autonomy	35.76	6.49	0.51**	0.32**	—					
4. Environmental mastery	27.60	5.04	0.80**	0.51**	0.59**	—				
5. Personal growth	32.58	5.19	0.49**	0.41**	0.28**	0.49**	—			
6. Composite psychological well-being score	151.50	21.46	0.85**	0.72**	0.72**	0.87**	0.68**	—		
7. Emotional exhaustion	17.35	11.58	−0.47**	−0.35**	−0.37**	−0.54**	−0.21**	−0.50**	—	
8. Personal accomplishment at work	32.03	7.58	0.44**	0.30**	0.33**	0.48**	0.41**	0.51**	−0.36**	—

Note: M, mean; SD, standard deviation. Correlation coefficients are presented below the diagonal. **p < 0.01.

Overall, participants reported relatively high levels of psychological well-being across all assessed dimensions and moderate levels of emotional exhaustion. The composite psychological well-being score was positively correlated with all five psychological well-being dimensions, with the strongest association observed for environmental mastery (*r* = 0.87, *p* < 0.01). All dimensions of psychological well-being were positively and significantly intercorrelated (all *p* < 0.01), with the strongest association observed between self-acceptance and environmental mastery. Furthermore, emotional exhaustion was negatively associated with all dimensions of psychological well-being and with the composite psychological well-being score, whereas personal accomplishment at work was positively associated with all dimensions and with the composite psychological well-being score. Environmental mastery showed the strongest associations with both burnout indicators, being negatively related to emotional exhaustion and positively related to personal accomplishment. Finally, emotional exhaustion was negatively correlated with personal accomplishment at work.

### Gender Differences in Psychological Well-Being

Gender differences in psychological well-being dimensions are presented in Table 3. No significant gender differences were observed in self-acceptance or environmental mastery. However, women reported significantly higher levels of positive relations and personal growth, whereas men reported significantly higher levels of autonomy.

**Table 3.** **Gender differences in psychological well-being dimensions**.

Dimension	Men M (SD)	Women M (SD)	*t*	*p*
Self-acceptance	27.88 (5.17)	27.77 (5.24)	0.25	0.804
Positive relations	27.07 (6.04)	28.39 (6.26)	−2.61	0.009
Autonomy	36.55 (5.87)	34.85 (6.97)	3.21	0.001
Environmental mastery	27.67 (4.86)	27.49 (5.26)	0.44	0.660
Personal growth	31.52 (4.89)	33.73 (5.29)	−5.28	<0.001

Note: M, mean; SD, standard deviation.

### Regression Analyses Predicting Burnout Indicators

#### Emotional Exhaustion

A multiple linear regression analysis was conducted to examine whether psychological well-being dimensions predicted emotional exhaustion (Table 4). The overall model was statistically significant and explained 31% of the variance in emotional exhaustion. Environmental mastery emerged as the strongest negative predictor of emotional exhaustion, followed by positive relations. In contrast, personal growth was positively associated with emotional exhaustion. Self-acceptance and autonomy were not significant predictors.

**Table 4.** **Multiple linear regression analysis predicting emotional exhaustion**.

Predictor	B	SE	β	95% CI for B	*p*
Intercept	52.101	3.000	—	[46.210, 57.993]	<0.001
Self-acceptance	−0.224	0.132	−0.101	[−0.483, 0.034]	0.089
Positive relations	−0.222	0.077	−0.118	[−0.373, −0.071]	0.004
Autonomy	−0.134	0.076	−0.075	[−0.282, 0.015]	0.077
Environmental mastery	−0.930	0.145	−0.405	[−1.215, −0.645]	<0.001
Personal growth	0.248	0.090	0.111	[0.071, 0.426]	0.006

Note: Dependent variable: Emotional exhaustion. Model statistics: R^2^ = 0.311, Adjusted R^2^ = 0.305, F(5, 591) = 53.352, *p* < 0.001. SE, standard error; CI, confidence interval.

#### Personal Accomplishment at Work

A second multiple linear regression analysis was conducted to examine whether psychological well-being dimensions predicted personal accomplishment at work (Table 5). The overall model was statistically significant and explained 28% of the variance in personal accomplishment. Environmental mastery and personal growth emerged as significant positive predictors. In contrast, self-acceptance, positive relations, and autonomy were not significant predictors. Overall, these findings indicate that faculty members with greater environmental mastery and personal growth tend to report higher levels of professional accomplishment.

**Table 5.** **Multiple linear regression analysis predicting personal accomplishment at work**.

Predictor	B	SE	β	95% CI for B	*p*
Intercept	4.237	2.010	—	[0.289, 8.184]	0.035
Self-acceptance	0.119	0.088	0.082	[−0.054, 0.292]	0.177
Positive relations	0.024	0.051	0.019	[−0.077, 0.125]	0.647
Autonomy	0.076	0.051	0.065	[−0.023, 0.176]	0.133
Environmental mastery	0.395	0.097	0.263	[0.204, 0.585]	<0.001
Personal growth	0.313	0.061	0.215	[0.194, 0.432]	<0.001

Note: Dependent variable: Personal accomplishment at work. Model statistics: R^2^ = 0.278, Adjusted R^2^ = 0.272, F(5, 591) = 45.496, *p* < 0.001. SE, standard error; CI, confidence interval.

To complement the dimensional regression analyses presented above, additional simple linear regression analyses were conducted using the composite psychological well-being score as the independent variable (Table 6). The composite psychological well-being score was significantly associated with both emotional exhaustion and personal accomplishment at work, supporting its use in the subsequent mediation analyses.

**Table 6.** **Simple linear regression analyses using the composite psychological well-being score as the independent variable**.

Outcome	B	SE	β	95% CI for B	*p*	R^2^
Emotional exhaustion	−0.271	0.019	−0.503	[−0.309, −0.234]	< 0.001	0.253
Personal accomplishment at work	0.178	0.012	0.505	[0.154, 0.203]	< 0.001	0.255

Note: Predictor: Composite psychological well-being score. Model statistics for emotional exhaustion: R^2^ = 0.253, Adjusted R^2^ = 0.252, F(1, 595) = 201.540, *p* < 0.001. Model statistics for personal accomplishment at work: R^2^ = 0.255, Adjusted R^2^ = 0.254, F(1, 595) = 203.811, *p* < 0.001. SE, standard error; CI, confidence interval.

### Mediation Analysis

For this analysis, psychological well-being was operationalized as the composite psychological well-being score, calculated by summing the scores of the five selected dimensions of Ryff’s Psychological Well-Being Scale (self-acceptance, positive relations, autonomy, environmental mastery, and personal growth).

**Fig. 1.**
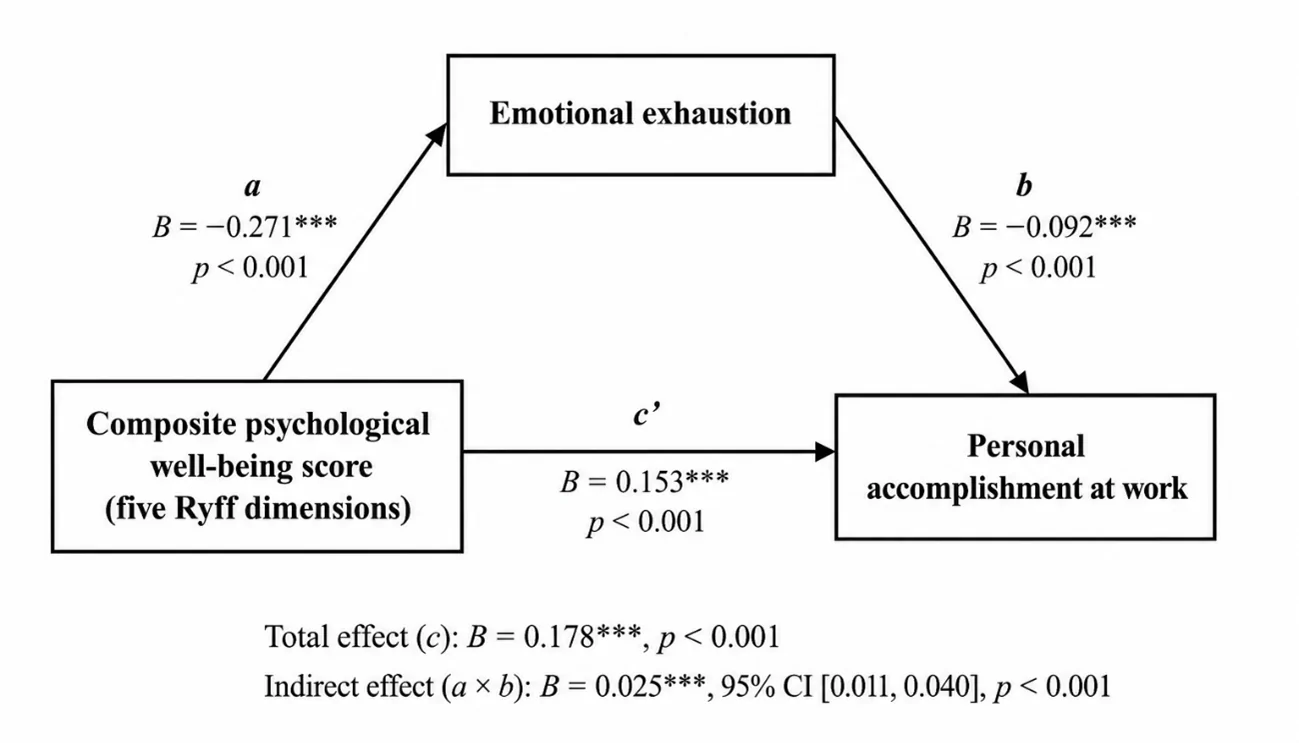
**Mediation model illustrating the indirect effect of the composite psychological well-being score on personal accomplishment at work through emotional exhaustion**. Note: The composite psychological well-being score was calculated by summing the scores of the five selected dimensions of Ryff’s Psychological Well-Being Scale (self-acceptance, positive relations, autonomy, environmental mastery, and personal growth). The total effect (*c*) was B = 0.178 (SE = 0.012, *p* < 0.001). The indirect effect (a × b) was B = 0.025 (SE = 0.007, 95% CI [0.011, 0.040], *p* < 0.001). CI, confidence interval.

A mediation analysis was conducted to examine whether emotional exhaustion mediated the relationship between the composite psychological well-being score and personal accomplishment at work (Fig. 1).

Results indicated a significant total effect of the composite psychological well-being score on personal accomplishment at work (B = 0.178, standard error (SE) = 0.012, *p* < 0.001). When emotional exhaustion was included as a mediator, the direct effect remained statistically significant (B = 0.153, SE = 0.014, *p* < 0.001), indicating partial mediation.

Furthermore, the indirect effect of the composite psychological well-being score on personal accomplishment through emotional exhaustion was also significant (B = 0.025, SE = 0.007, 95% confidence interval (CI) [0.011, 0.040], *p* < 0.001). Specifically, higher levels of the composite psychological well-being score were associated with lower emotional exhaustion, which in turn was related to greater personal accomplishment at work. Overall, these findings support the role of emotional exhaustion as a partial mediator in the relationship between the composite psychological well-being score and professional accomplishment among university faculty members.

## Discussion

The present study examined the relationship between psychological well-being and burnout dimensions among university faculty members, focusing on the predictive role of Ryff’s dimensions and the mediating effect of emotional exhaustion on professional accomplishment. Overall, higher psychological well-being was associated with lower emotional exhaustion and greater professional accomplishment, with environmental mastery emerging as the strongest predictor. Emotional exhaustion also partially mediated the relationship between psychological well-being and professional accomplishment, suggesting that emotional depletion may be an important factor statistically associated with the link between psychological resources and occupational functioning in university faculty.

These findings are consistent with previous research indicating that university teaching is characterized by high cognitive, emotional, and organizational demands [2, 6]. Contemporary academic environments require faculty members to simultaneously balance teaching, research productivity, publication pressure, administrative responsibilities, and institutional evaluation processes. Within the JD-R framework, prolonged exposure to these demands may contribute to emotional exhaustion when personal and organizational resources are insufficient [18, 25]. In this context, psychological well-being may operate as a relevant personal resource capable of buffering the negative effects of occupational stress [18, 26].

Correlation analyses revealed that all dimensions of psychological well-being were negatively associated with emotional exhaustion and positively associated with professional accomplishment. These findings are consistent with previous studies conducted in teachers and academic professionals, where greater well-being has been associated with lower burnout and better occupational adjustment [12, 14, 27]. Importantly, the present study supports Ryff’s multidimensional model as a useful framework for understanding occupational functioning in university faculty. Dimensions such as self-acceptance, autonomy, interpersonal functioning, and environmental mastery appear to represent adaptive psychological capacities that may facilitate resilience and professional sustainability in highly demanding academic settings.

Among all dimensions assessed, environmental mastery emerged as the strongest predictor of both emotional exhaustion and professional accomplishment. Faculty members reporting greater environmental mastery tended to experience substantially lower emotional exhaustion and higher levels of accomplishment at work. This finding is especially relevant because environmental mastery reflects the perceived ability to effectively manage responsibilities, organize environmental demands, and maintain control over complex situations [8, 9]. In university contexts characterized by workload overload, organizational pressure, and role conflict, this capacity may be particularly important for preserving emotional stability and professional functioning. Previous studies have similarly identified environmental mastery as one of the dimensions most strongly associated with adaptive coping and resilience in educational professionals [12, 13]. This interpretation is also consistent with the JD-R framework, which conceptualizes personal resources as psychological characteristics that facilitate adaptation to demanding occupational environments and help sustain effective functioning despite chronic job demands [15].

Interestingly, personal growth was positively associated with both professional accomplishment and emotional exhaustion. Although this pattern may appear paradoxical, it likely reflects the demanding nature of contemporary academic environments. Faculty members strongly oriented toward achievement, continuous development, and professional engagement may invest greater emotional and cognitive resources in their work, enhancing occupational accomplishment while also increasing vulnerability to emotional exhaustion. These findings suggest that some dimensions of psychological well-being may function as double-edged resources in highly demanding professional contexts [2, 14, 28]. One possible explanation is that faculty members with higher levels of personal growth may be more inclined to engage in professional development and other activities that foster competence and achievement [29]. Although such engagement may enhance feelings of competence and professional accomplishment, it may simultaneously increase workload, time pressure, and emotional investment. Consequently, personal growth may represent a beneficial psychological resource that is associated with greater professional accomplishment while also exposing individuals to higher levels of emotional exhaustion under demanding working conditions. This interpretation is consistent with previous research suggesting that highly engaged professionals may experience both enhanced occupational achievement and increased vulnerability to strain when sustained effort and involvement are not matched by sufficient recovery opportunities [3, 28].

The study also identified several sociodemographic differences in psychological well-being dimensions. Women reported higher levels of positive relations and personal growth, whereas men showed higher autonomy scores. Additionally, participants with stable partners or children tended to report greater self-acceptance and environmental mastery. Age and teaching experience were positively associated with self-acceptance, autonomy, and environmental mastery, while personal growth showed slight negative associations with both variables. These findings are generally consistent with developmental literature suggesting that emotional stability, self-regulation, and environmental control tend to increase with age and professional experience [8, 27, 30].

One of the most relevant findings concerns the mediating role of emotional exhaustion in the relationship between psychological well-being and professional accomplishment. Results indicated that emotional exhaustion partially mediated this relationship, suggesting that higher psychological well-being was associated with greater professional accomplishment and that this association was partially explained by lower levels of emotional exhaustion. These findings are consistent with previous mediation studies conducted in educational professionals, where psychological well-being and emotional regulation were associated with lower burnout and greater professional efficacy [12, 16, 17]. Also, these results are consistent with broader evidence suggesting that both personal and organizational resources may influence burnout through intermediate psychological processes involved in occupational adjustment. The present study extends previous literature by specifically examining emotional exhaustion as a mediator between Ryff’s multidimensional model and professional accomplishment in university faculty members.

From an applied perspective, the present findings highlight the importance of promoting psychological well-being within university environments to prevent burnout and sustain occupational functioning. Interventions focused on emotional regulation, adaptive coping, resilience, interpersonal support, and particularly environmental mastery may be associated with lower levels of emotional exhaustion among faculty members.

Likewise, organizational strategies aimed at reducing administrative burden, improving institutional support, and promoting work-life balance may contribute to healthier academic environments [4, 18, 31, 32], especially given the increasing prevalence of occupational distress among university staff in recent years [33, 34].

Several limitations should be acknowledged. First, the cross-sectional design prevents establishing causal relationships among variables. Second, all measures were based on self-report instruments, which may increase common method bias and social desirability effects. Third, participants were recruited using convenience sampling and most were affiliated with public Spanish universities, which may limit the generalizability of the findings. Finally, only two burnout dimensions were assessed, excluding depersonalization, which may also be relevant in university faculty adjustment.

## Conclusions

Overall, the present findings highlight psychological well-being as an important protective factor against burnout among university faculty members. Environmental mastery emerged as a key dimension associated with lower emotional exhaustion and greater professional accomplishment, while emotional exhaustion was statistically associated with the relationship between psychological well-being and professional accomplishment. These findings underscore the importance of promoting psychological well-being in academic settings as a potential strategy to prevent burnout and enhance professional functioning. Future longitudinal and cross-cultural studies are needed to confirm these relationships and evaluate the effectiveness of interventions aimed at strengthening psychological well-being among university faculty.

## Availability of Data and Materials

The datasets generated and/or analyzed during the current study are available from the corresponding author upon reasonable request.
